# The causal involvement of the BDNF-TrkB pathway in dentate gyrus in early-life stress-induced cognitive deficits in male mice

**DOI:** 10.1038/s41398-023-02476-5

**Published:** 2023-05-24

**Authors:** Ya-Xin Sun, Yun-Ai Su, Qi Wang, Jia-Ya Zheng, Chen-Chen Zhang, Ting Wang, Xiao Liu, Yu-Nu Ma, Xue-Xin Li, Xian-Qiang Zhang, Xiao-Meng Xie, Xiao-Dong Wang, Ji-Tao Li, Tian-Mei Si

**Affiliations:** 1 Peking University Sixth Hospital, Peking University Institute of Mental Health, NHC Key Laboratory of Mental Health (Peking University), National Clinical Research Center for Mental Disorders (Peking University Sixth Hospital), Beijing, 100191 China; 2 School of Mental Health, Wenzhou Medical University, Wenzhou, 325035 China; 3 Department of Neurobiology, Key Laboratory of Medical Neurobiology of Ministry of Health of China, Zhejiang Province Key Laboratory of Neurobiology, Zhejiang University School of Medicine, Hangzhou, 310058 China

**Keywords:** Depression, Molecular neuroscience

## Abstract

Cognitive dysfunction is a significant, untreated clinical need in patients with psychiatric disorders, for which preclinical studies are needed to understand the underlying mechanisms and to identify potential therapeutic targets. Early-life stress (ELS) leads to long-lasting deficits of hippocampus-dependent learning and memory in adult mice, which may be associated with the hypofunction of the brain-derived neurotrophic factor (BDNF) and its high-affinity receptor, tropomyosin receptor kinase B (TrkB). In this study, we carried out eight experiments using male mice to examine the causal involvement of the BDNF-TrkB pathway in dentate gyrus (DG) and the therapeutic effects of the TrkB agonist (7,8-DHF) in ELS-induced cognitive deficits. Adopting the limited nesting and bedding material paradigm, we first demonstrated that ELS impaired spatial memory, suppressed BDNF expression and neurogenesis in the DG in adult mice. Downregulating BDNF expression (conditional BDNF knockdown) or inhibition of the TrkB receptor (using its antagonist ANA-12) in the DG mimicked the cognitive deficits of ELS. Acute upregulation of BDNF (exogenous human recombinant BDNF microinjection) levels or activation of TrkB receptor (using its agonist, 7,8-DHF) in the DG restored ELS-induced spatial memory loss. Finally, acute and subchronic systemic administration of 7,8-DHF successfully restored spatial memory loss in stressed mice. Subchronic 7,8-DHF treatment also reversed ELS-induced neurogenesis reduction. Our findings highlight BDNF-TrkB system as the molecular target of ELS-induced spatial memory deficits and provide translational evidence for the intervention at this system in the treatment of cognitive deficits in stress-related psychiatric disorders, such as major depressive disorder.

## Introduction

Cognitive dysfunction has been increasingly recognized as one of the core deficits in several psychiatric disorders, such as major depressive disorder [1, 2]. As current therapeutic strategies have rather poor effects on cognitive impairment, it is urgent to identify the underlying molecular mechanisms and potential treatment targets so that effective treatments can be developed to address this far untreated clinical need [1, 2]. Adverse life experience during early neurodevelopmental periods is a well-recognized risk factor for several psychiatric disorders [3–5]. One consistent consequence of early-life stress (ELS) is cognitive deficits [6–10]. For instance, compared with control mice, mice exposed to ELS perform significantly worse in hippocampus-dependent learning and memory, such as in the spatial object recognition test [11–16]. Therefore, the ELS animal model provides a valuable tool for the treatment of cognitive deficits in psychiatric disorders.

Brain-derived neurotrophic factor (BDNF) and its high-affinity receptor, tropomyosin receptor kinase B (TrkB), are widely distributed in the central nervous system [17] and play important roles in neural development and function, including cell proliferation and differentiation, axon and dendrite growth, and synaptogenesis [18, 19]. The BDNF-TrkB signaling pathway contributes significantly to synaptic function and plasticity, including long-term potential [20, 21], which are crucial to cognitive functions [22–26]. Several lines of evidence support the crucial involvement of the BDNF-TrkB pathway in cognitive behaviors. Hippocampal-dependent learning and memory is impaired in mouse models of genetic mutations of BDNF [27, 28] or TrkB [29, 30]. Activating TrkB receptors by small-molecule agonists (e.g., 7,8-dihydroxyflavone, 7,8-DHF) improves cognition in normal rodents and animal models of cognitive deficits such as Alzheimer’s disease [26, 31–33]. The BDNF-TrkB pathway has also been reported to mediate cognition-enhancing effects of some drugs, e.g., (R)-ketamine [34, 35], memantine [36], melatonin [37, 38]. The therapeutic effects targeting at the BDNF-TrkB pathway in neurodevelopmental animal models have been seldom studied [39]. Several studies have consistently shown that ELS (mostly the maternal separation paradigm) downregulates BDNF protein and mRNA levels in hippocampus [40, 41]. One study also reported that 7,8-DHF intake via water could prevent deficits in novel object recognition and prepulse inhibition in an animal model of maternal immune activation [42]. However, it remains unclear whether and how the hippocampal BDNF-trkB pathway is causally involved in ELS-induced cognitive deficits.

In this study, we examined the causal role of the BDNF-TrkB pathway in the ELS-induced cognitive deficits and evaluated the therapeutic effects of 7,8-DHF, the TrkB agonist attracting widespread attention for its cognition-enhancing effects. We adopted the well-established ELS paradigm, the limited nesting and bedding material (LBN), which has been reported to induce hippocampal-dependent memory deficits [11, 43, 44] and neurogenesis alterations in dentate gyrus (DG), the hippocampal subregion that retains neurogenesis capacity in adult rodents. Eight experiments were carried out. We first examined the effects of the LBN paradigm on cognitive behaviors, BDNF expression, and neurogenesis in the DG (Exp. 1). We then examined the role of BDNF and TrkB in mediating ELS-induced cognitive impairment by specifically downregulating or upregulating BDNF levels (Exps. 2 and 3) or TrkB receptor function (Exps. 4 and 5) in the DG, resepectively. Finally, we examined whether acute and subchronic systemic administration of 7,8-DHF (Exps. 6–8) could restore ELS-induced cognitive deficits and/or neurogenesis reduction to provide translational evidence for the treatment of cognitive deficits in stress-related psychiatric disorders, such as major depressive disorder.

## Materials and methods

### Animals and housing

Adult male and female C57BL/6N mice (12 weeks old) were purchased from Vital River Laboratories (Beijing, China) for breeding. Every 2 females were housed with one male for 2 weeks and then housed separately. Females were checked at 9:00 a.m. and the day of delivery was marked as the postnatal day 0 (PND0). The *Bdnf*^tm2Jae^/J mice (Stock Number: 004339) were purchased from the Jackson Laboratory, possessing LoxP sites on either side of exon 5 of the BDNF gene [45], which maintained fully back-crossed onto C57BL/6N mice and adult male homozygous mice were used. Sample sizes in each group were chosen based on previous studies (*n* ≥ 8 for behavioral tests; *n* ≥ 4 for molecular tests).

All animals were housed under standard conditions (12:12 h light/dark cycle, lights on at 8:00, temperature 23 ± 1 °C, humidity 35%-55%) with no limitation to food or water. All procedures were performed in accordance with the National Institute of Health Guide for the Use and Care of Laboratory Animals and were approved by the Peking University Committee on Animal Care and Use.

### Early-life stress procedure

The early-life stress model induced by limited nesting and bedding material was established based on the previous studies of our laboratory [10, 11]. At 9:00–10:00 a.m. of PND2, pups were weighed and litters culled to six to eight pups with equal numbers of males and females whenever possible. Dams in the control group (*n* = 4, 7, 6, 6, 6 for experiment 1, 3, 5, 7, and 8, respectively) were provided with 4.8 g nesting material (2 squares of Nestlets, Ancare, New York, USA) and 500 ml of sawdust. In contrast, dams in the stress group (*n* = 4, 7, 8, 6, 7 for experiment 1, 3, 5, 7, and 8, respectively) were given a limited quantity of nesting material (1.2 g, 1/2 squares of Nestlets), placed on an aluminum mesh platform (26.5 cm × 15.5 cm; hole size: 0.5 cm × 1.0 cm; McNichols, Tampa, FL, USA) that was approximately 1.0 cm above the cage floor. All litters remained undisturbed during PND2–9. At 9:00–10:00 a.m. of PND9, the stress was removed and the stressed and non-stressed mothers raised their own offspring under standard bedding/nesting conditions until weaning. On PND28 the male pups were weaned, group-housed in 3–4 per cage, and used in the following experiments. Siblings were split into groups equally whenever possible, with 1–3 pups per dam in each group. The female pups were killed by decapitation. All experimental cages were refreshed once a week to keep clean with no further manipulation.

### Virus-mediated in vivo BDNF knockdown

Adult male *Bdnf*^tm2Jae^/J homozygous mice (8 weeks old) were anesthetized with isoflurane (3% induction, 1.5% maintenance) and received an anti-inflammatory medication (meloxicam; 0.5 mg/kg, i.p.). We bilaterally diffused AAV2/9-CaMKIIa-Cre-P2A-GFP and AAV2/9-CaMKIIa-GFP (Purchased from Vigenebio, Shandong) to the *Bdnf*^*tm2Jae*^*/*J mice to specifically downregulate *Bdnf* expression in DG granule neurons. We delivered 0.5 µl/hemisphere virus to the dorsal DG (0.18 cm posterior to bregma, 0.12 cm lateral from midline, 0.22 cm dorsoventral from the surface of skull) in 5 min and left micropipette for another 5 min for diffusion. Four weeks later, mice were tested for behavioral tests.

### Drug administration

For intraperitoneal administration (i.p.), 7,8-dihydroxyflavone (7,8-DHF; Catalog number: D5446, Sigma-Aldrich, USA, 5 mg/kg) was prepared in vehicle of 20% β-cyclodextrin in normal saline, and ANA-12 (Catalog number: HY-12497, MedChem Express, USA, 0.5 mg/kg) was prepared in vehicle of 20% β-cyclodextrin in normal saline. The doses of 7,8-DHF (5 mg/kg), and ANA-12 (0.5 mg/kg) were selected as reported previously [31, 46, 47].

For stereotaxic drug microinjection, Recombinant Human BDNF (rhBDNF; Catalog number: AF-450-02, Peprotech, USA, 0.5 μg/μl, 0.5 μl/site) was prepared in vehicle of 0.1% BSA, 7,8-DHF (1 μg/μl, 0.5 μl/site), and ANA-12 (1 μg/μl, 0.5 μl/site) was prepared in vehicle of 20% β-cyclodextrin in normal saline. The dose of 7,8-DHF was chosen based on our pilot study. The doses of BDNF [48], and ANA-12 [49] were selected as reported previously.

### Stereotaxic surgery and drug microinjection

Male C57BL/6J mice (8 weeks old) were anesthetized with isoflurane and received an anti-inflammatory and analgesic medication (meloxicam; 0.5 mg/kg, i.p.). The head of mice were then fixed in a stereotaxic frame (Reward; ShenZhen, China) where two holes were drilled at stereotaxic coordinates (AP −0.18 cm; ML ± 0.17 cm from bregma) on the surface of skull. Cannula (Cannula-Single/O.D.0.48mm-26G/M3.5; Reward, ShenZhen, China) was held by a clamper and was lowered through the holes bilaterally at a 9° angle until the cannula tips reached the DG (AP −0.18 cm; ML ±0.17 cm; DV −0.2 cm from bregma). Then we used dental acrylic cement and two small anchor screws to fix the cannula. Mice were put back to their home cages and were allowed to recover for 7 days, during which meloxicam was added to drinking water (0.5 ml meloxicam + 1 L drinking water), before being handled and taking behavioral tests.

### Behavioral testing

Behavioral tests were performed between 9:00 a.m. and 5:00 p.m. as previously described [10, 11]. The open field test was analyzed automatically by ANY-maze 4.98 (Stoelting, Wood Dale, IL, USA). The spatial object recognition task and Y-maze spontaneous alternation task were scored by an investigator blind to treatment conditions.

#### Open field

This test is used to assess animals’ anxiety-like behaviors and activity. Mice were placed in the open field arena (50 × 50 × 50 cm^3^) made of gray polyvinyl chloride and evenly illuminated at 60 lux. At the beginning of the test, the mice was placed in the corner of the arena and allowed to freely explore the apparatus for 10 min. The total distance traveled, time spent in the center zone, and the number of entering the center zone were analyzed.

#### Spatial object recognition task

The test is used to assess animals’ memory about object locations. The task was performed in the open field arena illuminated at 10 lux. Prominent spatial cues were provided. Mice were habituated to the arena for 10 min on 2 consecutive days before testing. The testing procedure included three consecutive sessions separated by intertrial intervals (ITIs) of 1 h. In two acquisition phases, mice were presented with two identical circular cones and allowed to freely explore for 10 min. During the 10-min retrieval trial, we moved one of the two object to a novel location (Displaced object), and the other one was kept in the old site (Stationary object). The time spent exploring each object was measured. The preference index (PI) was calculated as follows: PI = 100% × (time with the Displaced object)/time with both objects. Mice showing the total probe time with two objects in either acquisition or test phase below 10 s would be excluded from statistical analyses.

#### Y-maze spontaneous alternation test

This test is used to assess spatial working memory. Driven by the natural tendency for novelty, animals likely tend to alternate between the three arms in the Y-maze (termed as “spontaneous alternation”). Working memory is required in this process, as animals need to temporarily hold the memory of most recently visited arms [50–52]. The Y-maze apparatus was made of gray polyvinyl chloride with three symmetrical arms (30 × 10 × 15 cm^3^) and illuminated at 10 lux. During the test, mice were individually placed in the end of one arm and allowed to freely explore the arms for 5 min. The number of spontaneous alternations (SA: A → B → C), alternative arm returns (AAR: A → B → A) and same arm returns (SAR: A → A) were recorded manually by investigator. The total number of entries to three arms were also counted. The percentage of SA = 100% × (the number of SA)/(total arm entries − 2). The percentage of AAR and SAR = 100% × (the number of AAR or SAR)/total arm entries. The mice showing less than six entries would be excluded.

### Immunostaining and image analysis

Mice were anesthetized with sodium pentobarbital (200 mg/kg, i.p.) and transcardially perfused with 0.9% saline followed by 4% buffered paraformaldehyde. Following postfixation and cryoprotection, serial sections were prepared through the dorsal hippocampus (horizontal: Bregma −2.16 to −2.96 mm, in Experiment 1; coronal: Bregma −1.43 to −2.27 mm, in Experiment 2 and 8) and entorhinal cortex (horizontal: Bregma −2.36 to −4.12 mm, in Experiment 1) at 30 μm thickness and 180 μm intervals using a cryostat (Leica, Wetzlar, Germany). The following primary antibodies were used for immunostaining: rabbit anti-BDNF (1:1000 for immunohistochemistry, 1:500 for immunofluorescence; ab108319, Abcam, Cambridge, UK), rabbit anti-NGF (NGF; 1:1000; ab6199, Abcam, Cambridge, UK), rabbit anti-NT-3 (NT-3; 1:1000; 18084-1-AP, Proteintech, Chicago, USA), rabbit anti-Ki-67 (1:1000; ab15580, Abcam), and rabbit anti-DCX (1:5000; 4604S, Cell Signaling Technology).

For immunohistochemistry, free-floating sections were treated with 3% hydrogen peroxide (10 min) followed by 1% normal goat serum (1 h), and then they were labeled with primary antibodies overnight at 4 °C. The next day, after rinsing, sections were incubated with biotinylated secondary antibody (Zhongshan Golden Bridge Biotechnology, Beijing, China) for 2 h at room temperature. After rinsing, the 3,3′-Diaminobenzidine Horseradish Peroxidase Color Development Kit was used for staining. Finally, sections were transferred onto slides and coverslipped with neutral quick-drying adhesive.

For immunofluorescence, sections were treated with 1% normal donkey serum for 1 h and labeled with the rabbit anti-BDNF antibody overnight at 4 °C. The next day, sections were rinsed and labeled with Alexa Fluor 594-conjugated donkey anti-rabbit secondary antibody (1:500; Invitrogen, Carlsbad, CA, USA) for 2 h at room temperature. After rinsing, sections were transferred onto slides and coverslipped with Vectashield containing 4′,6-diamidino-2-phenylindole (Vector Laboratories, Burlingame, CA, USA).

To quantify the density of DCX^+^ and Ki-67^+^ cells in dorsal DG (Fig. S1A), images from 3–4 sections (both hemispheres, 180 μm apart) per animal were acquired at 400× microscope (40× objective, NA 0.95) using the Olympus VS200 virtual slide scanning system. Positive cells were counted manually by an investigator blind to the experimental conditions. The length of granule cell layer (GCL) was measured using ImageJ. The density of positive cells in each hemisphere of a section was calculated as the number of positive cells divided by the GCL length. The cell density was then averaged across hemispheres and sections for each animal.

To quantify the immunoreactivity of BDNF, NGF, and NT-3, images (1280 × 960 pixel^2^) from 3–4 sections (both hemispheres, 180 μm apart) per animal were acquired at 100× using the Olympus BX51 microscope (Olympus, Tokyo, Japan) fitted with a CoolSNAP MP5 CCD camera (Roper Scientific) and were analyzed by ImageJ as described previously [53]. Relative protein levels were determined by the mean differences in optical density values between the regions of interest (Fig. S1B) and the corpus callosum (which generally lacks staining and was considered as background) from the same slide.

For colocalization analysis, images (1024 × 1024 pixel^2^) were obtained with an Olympus IX81 laser-scanning confocal microscope (Olympus, Tokyo, Japan) at 200× magnification using the Kalman filter and sequential scanning mode under identical settings for laser power, photomultiplier gain and offset. Images were adjusted for optimal brightness and contrast using the FV10-ASW 1.7 Software (Olympus).

### Western blot

Mice were anesthetized by isoflurane-O2 (4–5:100) at 24 h after the last behavioral test. Following the previously reported procedure [11, 54], we removed animal brains rapidly and dissected DG. DG samples from both hemispheres were homogenized in ice-cold lysis buffer and centrifuged at 12,000 rpm at 4 °C (10 min × 2). Protein concentrations were determined using a bicinchoninic acid protein assay kit (Pierce, Rockford, IL, USA). Samples containing 20 µg of protein were resolved by 10% sodium dodecyl sulfate-polyacrylamide gels, and transferred onto polyvinylidene difluoride membranes (Millipore, Bedford, MA, USA). Membranes were labeled with primary antibodies at 4 °C (overnight). The following antibodies were used: rabbit anti-BDNF (1:1000, ab108319, Abcam), and rabbit anti-GAPDH (1:20,000, 2118, Cell Signaling). After incubation with horseradish peroxidase-conjugated secondary antibodies (1:5000–20,000, Zhongshan Gold Bridge Biotechnology, China, diluted in TBST) at room temperature (2 h), bands were visualized using the Amersham Imager 600 (GE Healthcare, PA) and analyzed using Quantity One 4.2 (Bio-Rad, Hercules, CA) by an investigator blind to the treatment conditions. The values were corrected based on their corresponding control protein levels. All results were normalized by taking the value of the vehicle group as 100%.

### Statistical analysis

SPSS 26.0 (SPSS, Chicago, IL, USA) was used to perform statistical analyses. Comparisons between two groups were analyzed by Student’s *t* test (with equal variance) or Welch’s *t*-test (with unequal variance). BDNF, NGF, and NT-3 protein levels were analyzed by two-way analysis of variance (ANOVA) (stress × subregion). The effects of ELS and 7,8-DHF treatment on cognitive behaviors and neurogenesis were analyzed using two-way ANOVA (stress × treatment). When two-way ANOVA yielded a significant interaction, we carried out Tukey’s *post hoc* test to examine group differences in detail. For SOR tasks, one-sample *t* test was used to compare preference index with 0.5. Values significantly higher than 0.5 would indicate more exploration of the displaced object. Data are reported as mean ± SEM. Statistical significance was defined at two-sided *p* < 0.05.

## Results

### Early-life stress impairs spatial memory and reduces neurogenesis and DG BDNF expression in adult mice

**Fig. 1:**
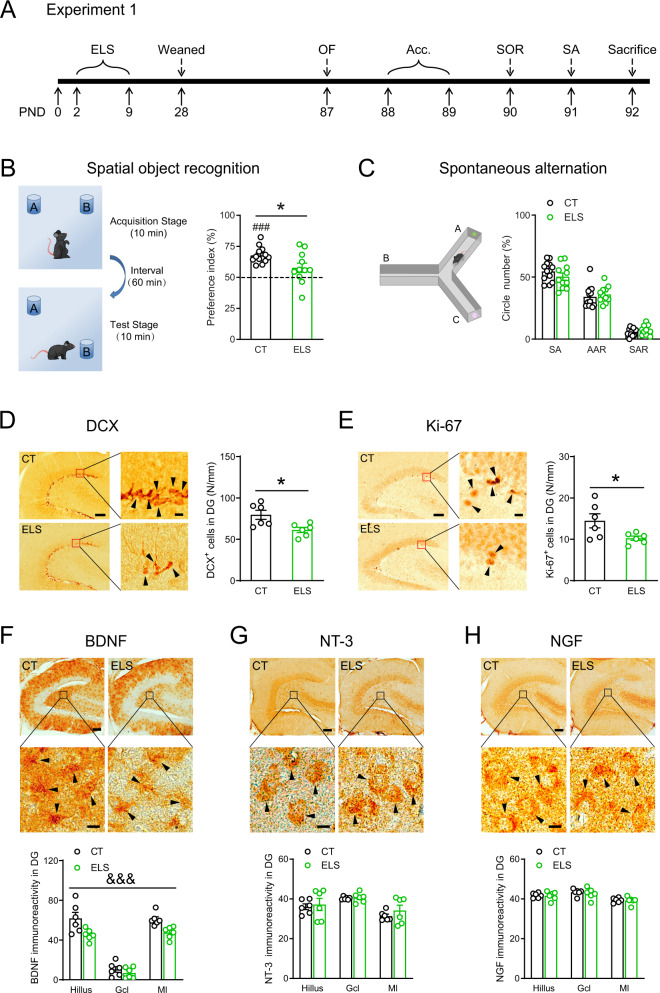
Effects of early-life stress on cognition, hippocampal neurogenesis, and neurotrophin levels in the dentate gyrus in adult mice. **A** The experimental timeline of the behavioral procedure and brain tissue acquisition after stress exposure. **B** In the spatial object recognition task, control mice distinguished the displaced object from the non-displaced one, whereas stressed mice failed to do so and performed worse than the controls. **C** ELS did not affect the performance in the Y-maze spontaneous alternation test. The number of DCX-positive (**D**) and Ki-67-positive (**E**) neurons in the DG (arrowheads) was decreased in the stressed mice. Scale bar = 100 µm or 10 µm. Immunostaining revealed that ELS significantly reduced the protein levels of BDNF (**F**), not NT-3 (**G**) or NGF (**H**), in the DG in adult mice. Representative images in (**D**–**H**) were captured using the Olympus VS200 virtual slide scanning system. Arrowheads, positive neurons; scale bar = 100 µm or 30 µm. AAR alternate arm return, Acc. acclimation, BDNF brain-derived neurotrophic factor, CT control, DCX doublecortin, DG dentate gyrus, ELS early-life stress, gcl granule cell layer, ml molecular layer, NGF neurotrophin nerve growth factor, NT-3 neurotrophin 3, OF open field, PND postnatal day, SA spontaneous alternation, SAR same arm return, SOR spatial object recognition. *p < 0.05, unpaired *t* test; ^###^*p* < 0.001, one-sample *t* test; ^&&&^*p* < 0.001, the main effect of stress.

To evaluate the impact of early-life stress on cognition in adult mice, we carried out the hippocampus-dependent spatial object recognition (SOR) and Y-maze spontaneous alternation tasks (Fig.1A). In the SOR test (Fig. 1B), unlike control mice (*t*_12_ = 9.957, *p* < 0.0001, *n* = 13, one-sample *t* test), mice in the ELS group only showed a trend to discriminate the displaced object from the non-displaced one (*t*_10_ = 2.053, *p* = 0.067, *n* = 11, one-sample *t* test) and exhibited significantly lower preference index than the CT group (*t*_22_ = 2.522, *p* = 0.019; unpaired *t* test), indicating that ELS induces spatial memory deficits. The total exploration time and the total distance traveled did not significantly differ between groups during the acquisition phase of this task (Fig. S2A). Spatial working memory was not affected by ELS, as two groups showed similar SA, AAR, and SAR in the Y-maze spontaneous alternation task (*p*s > 0.240, Fig. 1C). Note that ELS significantly increased the total number of entries to three arms (*t*_22_ = 2.353, *p* = 0.028; CT: *n* = 13, ELS: *n* = 11, unpaired *t* test, Fig. S2B), indicative of increased exploration in Y-maze. Two groups of mice exhibited similar anxiety-like behaviors in the open field arena (Fig. S2C).

To investigate the effects of ELS on adult hippocampal neurogenesis, Ki-67 (a marker for proliferation) and DCX (a specific marker for early phases of neurogenesis) were immunostained to quantify proliferating cells and young neurons. We found that ELS significantly reduced the density of DCX^+^ neurons (*t*_10_ = 2.904, *p* = 0.016; *n* = 6 per group, unpaired *t* test, Fig. 1D) and Ki-67^+^ neurons (*t*_10_ = 2.352, *p* = 0.041; *n* = 6 per group, unpaired *t* test, Fig. 1E) in DG.

We then examined whether ELS affects the expression levels of BDNF in DG on PND90 (Fig. 1F–H). Two-way ANOVA revealed a significant main effect of ELS on BDNF expression, with ELS-treated mice showing significantly lower BDNF levels (*F*_1, 30_ = 16.61, *p* = 0.0003, Fig. 1F). No ELS effects were found for NT-3 (*F*_1, 30_ = 0.880, *p* = 0.356, Fig. 1G) or NGF (*F*_1, 30_ = 0.844, *p* = 0.366, Fig. 1H) levels. Besides DG, we also explored BDNF expression in other hippocampus-related regions and found that ELS led to significant BDNF reduction in CA3 (*F*_1, 50_ = 29.90, *p* < 0.0001, Fig. S2D) and a trend of reduction in CA1 (*F*_1, 40_ = 3.248, *p* = 0.079, Fig. S2E) and that no ELS effects were observed in entorhinal cortex (*F*_1, 20_ = 0.360, *p* = 0.555, Fig. S2F).

### BDNF in DG mediated early-life stress-induced spatial memory impairment

**Fig. 2:**
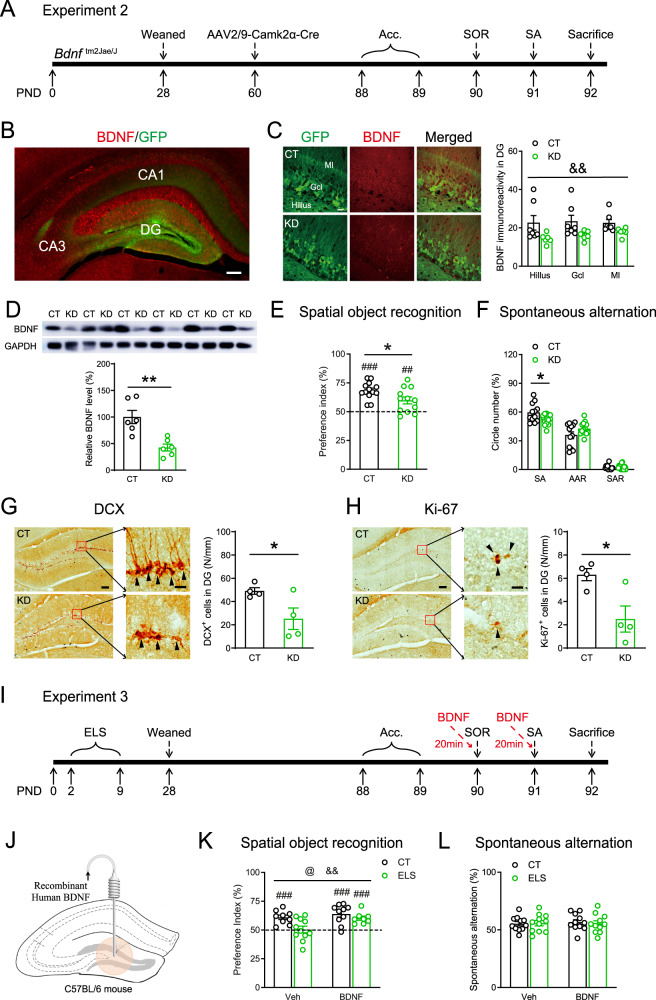
BDNF in the DG mediated early-life stress-induced spatial memory impairment. **A** The experimental timeline of the behavioral procedure and brain tissue acquisition after viral injection. **B** Region-specific expression of GFP in the DG is shown. Scale bar = 200 µm. **C** Representative images showing the expression of BDNF and GFP in the DG of CT and KD mice. Immunostaining analyses confirmed knockdown-induced reduction of BDNF expression in the DG. Scale bar = 20 µm. **D** Western blot analyses confirmed the knockdown-induced reduction of BDNF expression in the DG. **E** In the SOR test, although both CT and KD mice showed preference to the novel location, CT mice had higher preference index than KD mice. **F** KD mice had a lower spontaneous alternation ratio in the Y-maze test than control mice. BDNF knockdown decreased the number of DCX-positive (**G**) and Ki-67-positive (**H**) neurons in the DG (arrowheads). Scale bar = 100 µm or 20 µm. **I** The experimental timeline of the behavioral procedure and rhBDNF infusion after ELS exposure. **J** Schematic showing the DG local injection of rhBDNF in adult C57BL/6N mice. **K** In the SOR test, except that the ELS + Veh group failed to recognize the object placed in novel location, the other three groups performed well. **L** ELS or rhBDNF did not affect the spontaneous alternation ratio in the Y-maze test. AAR alternate arm return, Acc. acclimation, BDNF brain-derived neurotrophic factor, CT control, DCX doublecortin, DG dentate gyrus, ELS early-life stress, gcl granule cell layer, ml molecular layer, KD knockdown, OF open field, PND postnatal day, SA spontaneous alternation, SAR same arm return, SOR spatial object recognition, Veh vehicle. *p < 0.05, unpaired *t* test; ^##^*p* < 0.01^, ###^*p* < 0.001, one-sample *t* test; ^&&^*p* < 0.01, the main effect of virus.

To examine whether BDNF in DG is causally involved in ELS-induced cognitive deficits, we first evaluated the impact of downregulating BDNF expression in dorsal DG on spatial memory in adult mice. After we injected the AAV2/9-CaMKIIa-Cre-P2A-GFP virus into the DG of adult male *Bdnf*^tm2Jae/J^ mice to specifically knock down BDNF levels in the DG, we carried out SOR and Y-maze tasks (Fig. 2A, B). Compared with control mice, BDNF-KD mice showed significantly lower BDNF immunoreactivity in the DG (*F*_1, 33_ = 11.22, *p* = 0.002, Fig. 2C). Western blot analyses further confirmed the reduction of BDNF in the DG (*t*_10_ = 4.058, *p* = 0.002; *n* = 6 per group, unpaired *t* test, Fig. 2D). In SOR (Fig. 2E), while both KD and control mice successfully recognized the object placed in novel location (CT: *t*_12_ = 8.975, *p* < 0.0001, *n* = 13; KD: *t*_11_ = 3.227, *p* = 0.0081, *n* = 12; one-sample *t* test), KD mice showed significant decrease in preference index compared with the CT mice (*t*_23_ = 2.365, *p* = 0.027; unpaired *t* test). No group differences were found in the total probe time or total distance traveled during the acquisition stage (Fig. S3A). In the Y-maze task (Fig. 2F), BDNF knockdown significantly impaired spatial working memory, reflected by decreased SA ratio (*t*_24_ = 2.388, *p* = 0.025; CT: *n* = 12, KD: *n* = 14, unpaired *t* test) and a trend of increased AAR (*t*_24_ = −1.773, *p* = 0.089). The SAR or the total number of entries (Fig. S3B) were not significantly affected (*ps* > 0.640; unpaired *t* test). Similar with ELS, BDNF knockdown also significantly reduced the density of DCX^+^ neurons (*t*_6_ = 2.461, *p* = 0.049; *n* = 4 per group, unpaired *t* test, Fig. 2G) and Ki-67^+^ neurons (*t*_6_ = 3.087, *p* = 0.022; *n* = 4 per group, unpaired *t* test, Fig. 2H). Taken together, BDNF knockdown in DG reproduces the effects of early-life stress on spatial memory and neurogenesis.

Next, we examined whether local injection of recombinant human BDNF (rhBDNF) in DG could restore spatial memory deficits of early-life stressed mice. After 7-day recovery from stereotaxic surgery of cannula implantation, mice were tested in the SOR and Y-maze tests after 20-min infusion of rhBDNF or phosphate buffered saline (Fig. 2I, J). In the SOR task (Fig. 2K), except that the ELS + Veh group failed to recognize the object placed in novel location (*t*_10_ = 0.140, *p* = 0.892, *n* = 11, one-sample *t* test), the other three groups performed well (CT + Veh: *t*_8_ = 5.630, *p* < 0.001, *n* = 9; CT + BDNF: *t*_9_ = 5.250, *p* < 0.001, *n* = 10; ELS + BDNF: *t*_7_ = 5.768, *p* < 0.001, *n* = 8; one-sample *t* test), suggesting that BDNF elevation in DG restored ELS-induced cognitive deficits. Two-way ANOVA analysis on the preference index (Fig. 2K) revealed significant main effects of ELS (*F*_1, 34_ = 8.126, *p* = 0.007) and rhBDNF (*F*_1, 34_ = 6.537, *p* = 0.015), although the ELS × rhBDNF interaction did not approach significance (*F*_1, 34_ = 1.920, *p* = 0.175). During the acquisition stage (Fig. S3C), there were no main effects of rhBDNF in the total probe time or total distance traveled (*p*s > 0.437), while ELS significantly increased the total distance traveled (*F*_1, 34_ = 4.585, *p* = 0.040), without affecting the total probe time (*p* > 0.582). In the Y-maze test (Fig. 2L and Fig. S3D), similar with Exp.1, ELS did not significantly alter spatial working memory as measured by SA, AAR, or SAR (*p*s > 0.133). The main effect of rhBDNF was only found in SAR in that rhBDNF significantly reduced SAR ratio (*F*_1, 40_ = 6.849, *p* = 0.013), indicative of spatial working memory improvement.

By down- or upregulating BDNF levels in DG, our results in Exp. 2 and 3 indicate that BDNF is causally involved in ELS-induced spatial memory deficits.

### TrkB receptor in DG mediated early-life stress-induced spatial memory loss

To further investigate how TrkB receptor mediates early-life stress-induced spatial memory loss, we examined the effects of local infusion of the TrkB receptor antagonist (ANA-12) and agonist (7,8-DHF) in DG on cognitive behaviors.

**Fig. 3:**
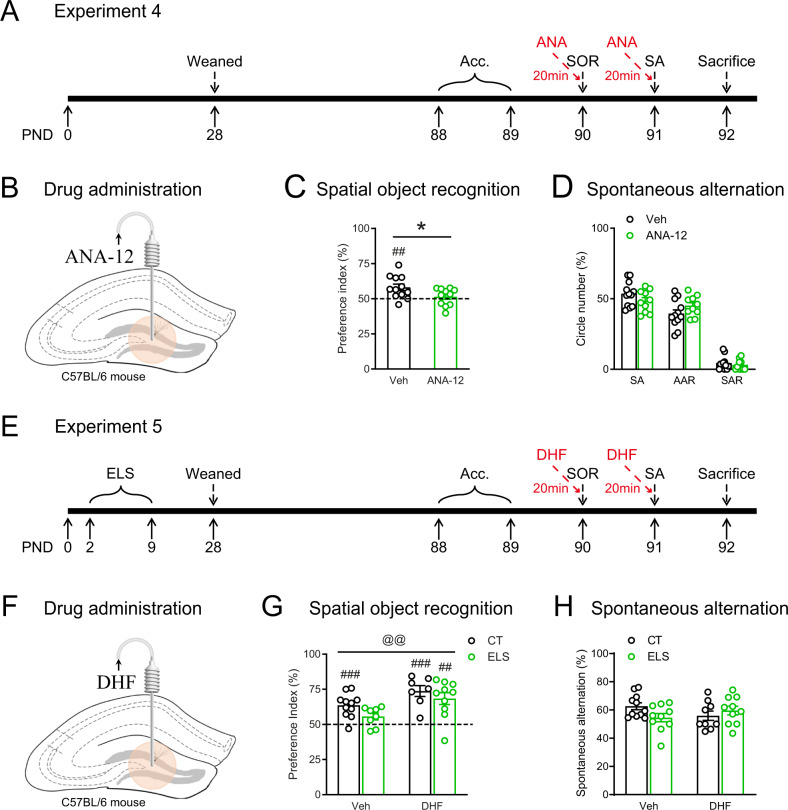
TrkB receptor in the DG mediated early-life stress-induced spatial memory loss. **A** The experimental timeline of the behavioral procedure and ANA-12 local injection. **B** Schematic showing the local infusion of ANA-12 in DG in adult C57BL/6N mice. **C** Compared with control mice that successfully distinguished the displaced object from the stationary one, ANA-12-treated mice showed impaired spatial recognition memory. **D** In the Y-maze spontaneous alternation task, ANA-12 infusion did not significantly affect spatial working memory. **E** The experimental timeline of the behavioral procedure and 7,8-DHF microinjection after ELS exposure. **F** Schematic showing the 7,8-DHF infusion into the DG in adult C57BL/6N mice. **G** In the SOR test, except that the ELS + Veh group failed to recognize the object placed in novel location, the other three groups performed well. **H** In the Y-maze test, microinjection of 7,8-DHF in DG did not significantly affect spatial working memory. AAR alternate arm return, Acc. acclimation, CT control, DHF 7,8-dihydroxyflavone, DG dentate gyrus, ELS early-life stress, PND postnatal day, SA spontaneous alternation, SAR same arm return, SOR spatial object recognition, Veh vehicle. *p < 0.05, unpaired *t* test; ^##^*p* < 0.01^, ###^*p* < 0.001, one-sample *t* test.

First, we examined the effects of local infusion of ANA-12 (1 μg/μl, 0.5 μl/site, 20 min before tests, Fig. 3A, B) on cognitive behaviors. In the SOR test (Fig. 3C and Fig. S4A), while the control mice distinguished the displaced object from the stationary one (*t*_11_ = 3.505, *p* = 0.005, *n* = 12, one-sample *t* test), the ANA-treated mice failed to do so (*t*_10_ = 0.768, *p* = 0.460, *n* = 11, one-sample *t* test). Compared with vehicle, ANA-12 infusion significantly decreased the preference index (*t*_21_ = 2.218, *p* = 0.038; unpaired *t* test), suggesting that inhibition of TrkB receptor mimicked the cognition-impairing effects of ELS. In the Y-maze test (Fig. 3D and Fig. S4B), microinjection of ANA-12 in DG did not significantly affect spatial working memory.

Next, we administrated 7,8-DHF, the TrkB receptor agonist, by intra-DG microinjection (1 µg/µl, 0.5 μl/site, 20 min before the test, Fig. 3E, F) to see whether the negative effects of stress could be reversed. In the SOR test (Fig. 3G and Fig. S4C), while the ES + Veh group only showed a trend to recognize the object placed in novel location (*t*_7_ = 2.301, *p* = 0.055, *n* = 8, one-sample *t* test), the other three groups performed well (CT + Veh: *t*_10_ = 5.347, *p* < 0.001, *n* = 11; CT + DHF: *t*_6_ = 6.075, *p* < 0.001, *n* = 7; ELS + DHF: *t*_9_ = 4.389, *p* = 0.002, *n* = 10; one-sample *t* test). Two-way ANOVA (Fig. 3G) revealed a tendency of ELS (*F*_1, 32_ = 3.812, *p* = 0.060) to reduce preference index and significant effects of 7,8-DHF to improve preference index (*F*_1, 32_ = 11.22, *p* = 0.002), without ELS × DHF interaction (*F*_1, 32_ = 0.146, *p* = 0.705). ELS or 7,8-DHF did not affect spatial working memory in the Y-maze test (Fig. 3H and Fig. S4D; *p*s > 0.091). These results suggest that microinjection of 7,8-DHF in DG reversed the negative effects of ELS on spatial memory in the SOR test.

### Acute systemic administration of 7,8-DHF partially reversed stress-induced spatial memory loss

Having shown that the BDNF-TrkB pathway in DG is causally involved in ELS-induced cognitive deficits, we then evaluated the potential therapeutic effects when targeting at the pathway, by systemic administration of TrkB receptor antagonist (ANA-12) and agonist (7,8-DHF). We first examined the acute effects of these drugs by intraperitoneal injection 60-min before behavioral tests.

**Fig. 4:**
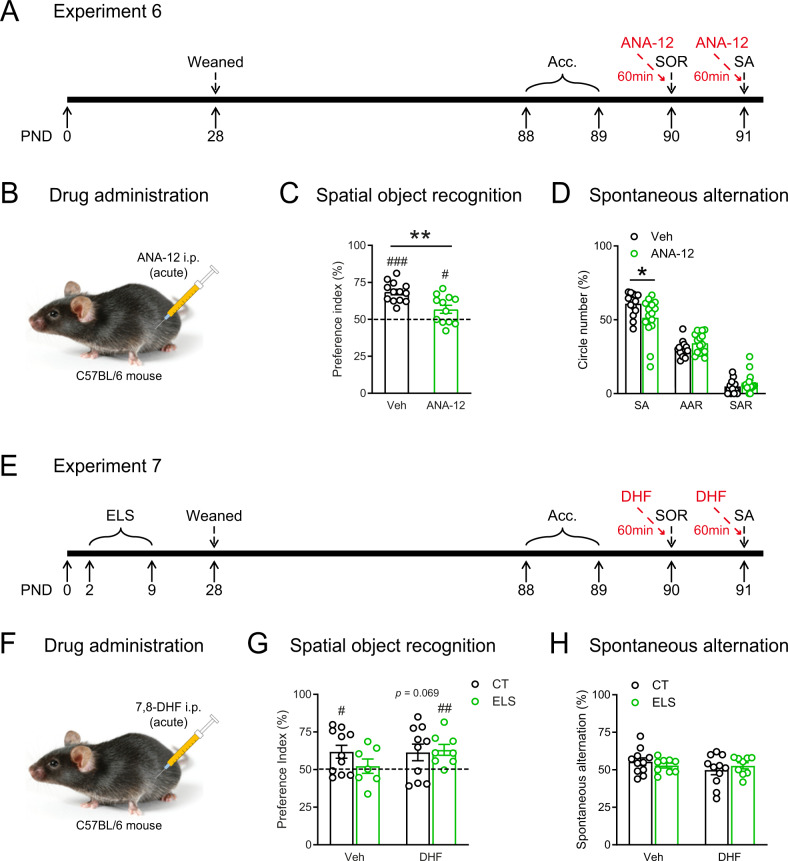
Acute systemic administration of 7,8-DHF partially reversed stress-induced spatial memory loss. **A** The experimental timeline of the behavioral procedure and ANA-12 injection. **B** Schematic showing the acute intraperitoneal injection of ANA-12 into adult C57BL/6 N mice. **C** In the SOR test, although both CT and ANA-12-treated mice showed preference to the novel location, CT mice had higher preference index than ANA-12-treated mice. **D** ANA-12-treated mice had a lower spontaneous alternation ratio in the Y-maze test than control mice. **E** The experimental timeline of the behavioral procedure and 7,8-DHF injection after ELS exposure. **F** Schematic showing the acute intraperitoneal injection of 7,8-DHF into adult C57BL/6 N mice. **G** In the SOR test, except that the ELS + Veh group failed to recognize the object placed in novel location, the other three groups performed well. **H** In the Y-maze test, microinjection of 7,8-DHF in DG did not significantly affect spatial working memory. AAR alternate arm return, Acc. acclimation, CT control, DHF 7,8-dihydroxyflavone, ELS early-life stress, PND postnatal day, SA spontaneous alternation, SAR same arm return, SOR spatial object recognition, Veh vehicle. *p < 0.05, **p < 0.01, unpaired *t* test; ^#^*p* < 0.05, ^##^*p* < 0.01^, ###^*p* < 0.001, one-sam*p*le *t* test.

First, acute ANA-12 treatment (0.5 mg/kg, Fig. 4A, B) was carried out to examine whether TrkB receptor inhibition could impair spatial memory. In the SOR test (Fig. 4C), while both control and ANA-treated mice successfully recognized the object placed in novel location (CT: *t*_12_ = 9.848, *p* < 0.0001, *n* = 13; ANA-12: *t*_11_ = 2.492, *p* = 0.030, *n* = 12; one-sample *t* test), ANA-12 significantly decreased preference index compared with the control group (*t*_23_ = 3.583, *p* = 0.002; unpaired *t* test). No group differences were observed for the total exploration time and distance during the acquisition stage (Fig. S5A). In the Y-maze test (Fig. 4D and Fig. S5B), ANA-12 significantly decreased SA (*t*_27_ = 2.262, *p* = 0.032; Veh: *n* = 14, ANA-12: *n* = 15, unpaired *t* test), and did not affect AAR (*p* = 0.122) or SAR (*p* = 0.238). These results indicate that blocking TrkB receptor acutely induced spatial memory loss, which are largely consistent with our previous experiments of downregulating the BDNF-TrkB pathway, i.e., by BDNF knockdown and local infusion of ANA-12 in DG.

Next, we administrated 7,8-DHF i.p. (5 mg/kg, Fig. 4E, F) to examine whether ELS-induced cognitive deficits could be reversed. In the SOR test (Fig. 4G and Fig. S5C), the ES + Veh group was the only group that failed to recognize the object placed in novel location (*t*_6_ = 0.486, *p* = 0.644, *n* = 7, one-sample *t* test), whereas the other three groups distinguished the displaced object from the stationary one either significantly or with a trend to reach significance (CT + Veh: *t*_10_ = 2.764, *p* < 0.001, *n* = 11; CT + DHF: *t*_9_ = 2.065, *p* = 0.069, *n* = 9; ELS + DHF: *t*_7_ = 3.597, *p* = 0.009, *n* = 8; one-sample *t* test). Two-way ANOVA on preference index revealed no significant effects of ELS (*F*_1, 32_ = 0.672, *p* = 0.419), 7,8-DHF (*F*_1, 32_ = 1.152, *p* = 0.291), or their interaction (*F*_1, 32_ = 1.343, *p* = 0.255). These results indicate that acute systemic administration of 7,8-DHF partially reversed ELS-induced spatial memory loss. ELS or 7,8-DHF did not affect spatial working memory in the Y-maze test (Fig. 4H and Fig. S5D; *p*s > 0.067). Interestingly, similar with Exp.1, ELS was also found to significantly increase the total number of entries in three arms (*F*_1, 36_ = 4.590, two-way ANOVA, *p* = 0.039, Fig. S5D).

### Subchronic systemic administration of 7,8-DHF reversed ELS-induced spatial memory deficits and adult neurogenesis reduction

**Fig. 5:**
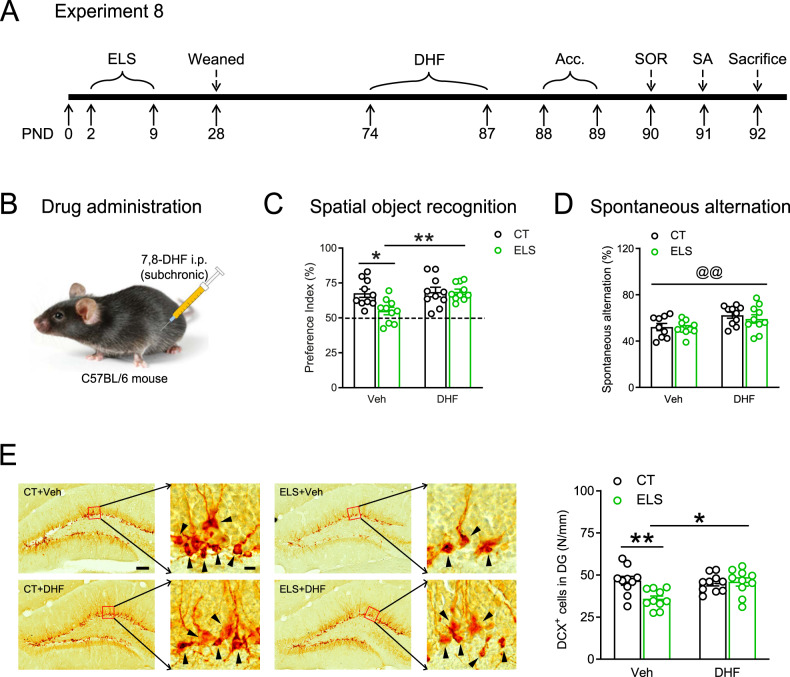
Subchronic systemic administration of 7,8-DHF reversed ELS-induced spatial memory deficits and adult neurogenesis reduction. **A** The experimental timeline of the 7,8-DHF injection and behavioral procedure after stress exposure. **B** Schematic showing the subchronic intraperitoneal injection of 7,8-DHF into adult C57BL/6N mice. **C** In the SOR test, except that the ELS + Veh group failed to recognize the object placed in novel location, the other three groups performed well. **D** In the Y-maze test, 7,8-DHF treatment increased the spontaneous alternation ratio. **E** Stress-induced reduction of the number of DCX-positive cells (arrowheads) in the DG was reversed by subchronic 7,8-DHF treatment. Scale bar = 100 µm or 10 µm. AAR alternate arm return, Acc. acclimation, CT control, DHF 7,8-dihydroxyflavone, ELS early-life stress, PND postnatal day, SA spontaneous alternation, SAR same arm return, SOR spatial object recognition, Veh vehicle. *p < 0.05, **p < 0.01, Tukey’s *post hoc* test; ^@^*p* < 0.05, ^@@^*p* < 0.01^,^ the main effect of drug.

Finally, we examined whether subchronic administration of 7,8-DHF (5 mg/kg, 14 days, Fig. 5A, B) could reverse ELS-induced cognitive deficits and neurogenesis reduction several days after the last drug treatment.

In the SOR test (Fig. 5C), except that the ES + Veh group failed to recognize the object placed in novel location (*t*_9_ = 1.847, *p* = 0.098, *n* = 10, one-sample *t* test), the other three groups performed well (CT + Veh: *t*_9_ = 5.914, *p* < 0.001; CT + DHF: *t*_9_ = 5.412, *p* < 0.001; ELS + DHF: *t*_9_ = 9.702, *p* < 0.001; *n* = 10 per group, one-sample *t* test). Two-way ANOVA (Fig. 5C) on preference index showed significant main effects of stress (*F*_1, 36_ = 4.984, *p* = 0.032), DHF (*F*_1, 36_ = 6.799, *p* = 0.013), and their interaction (*F*_1, 36_ = 5.253, *p* = 0.028). Post hoc comparisons showed that activation of TrkB receptor ameliorated ELS-induced deficits in SOR (ELS + Veh vs. ELS + DHF: *p* = 0.007). Neither the total probe time nor total distance traveled were influenced by intraperitoneal injection of 7,8-DHF at the acquisition stage (Fig. S6A).

In the Y-maze test, there were no significant main effects of ELS or stress × drug interaction (*p*s > 0.243). Main effects of 7,8-DHF were observed in SA (*F*_1, 35_ = 9.782, *p* = 0.004, Fig. 5D) and SAR (*F*_1, 35_ = 6.423, *p* = 0.016, Fig. S6B right), as 7,8-DHF significantly increased SA and decreased SAR. Interestingly, the ELS-induced increases in the total number of entries were reversed by subchronic treatment of 7,8-DHF (stress × drug interaction: *F*_1, 35_ = 14.12, *p* < 0.001; main effect of stress: *F*_1, 35_ = 2.805, *p* = 0.103; main effect of drug: *F*_1, 35_ = 4.019, *p* = 0.053; ELS + Veh vs. ELS + DHF: *p* = 0.001; *n* = 10 per group; Fig. S6B left).

We also examined the effects of subchronic 7,8-DHF treatment on ELS-induced neurogenesis reduction. Several days after the last treatment, 7,8-DHF still successfully reversed the decreased number of DCX^+^ neurons caused by ELS (Fig. 5E). Two-way ANOVA revealed significant stress × drug interaction (*F*_1, 36_ = 7.119, *p* = 0.011), main effect of stress (*F*_1, 36_ = 5.490, *p* = 0.025), and main effect of drug (*F*_1, 36_ = 4.107, *p* = 0.050). *Post hoc* analyses revealed that ELS significantly reduced the number of DCX^+^ neurons in vehicle-treated mice (CT + Veh vs. ELS + Veh: *p* = 0.006, *n* = 10 per group) and that 7,8-DHF treatment significantly increased the number of DCX^+^ neurons in stressed mice (ELS + Veh vs. ELS + DHF: *p* = 0.011, *n* = 10 per group).

## Discussion

In this study, we examined the causal role of the BDNF-TrkB pathway in DG in ELS-induced cognitive deficits in adult male mice. Our results demonstrate that exposure to adverse life experiences during the first postnatal two weeks decreased BDNF levels and neurogenesis in DG, accompanied by spatial memory deficits in the spatial object recognition test. Cognitive deficits were reproduced in experiments of downregulating the BDNF-TrkB pathway function by BDNF knockdown in the DG or inhibition of TrkB receptor via ANA-12. Importantly, ELS-induced cognitive deficits were rescued by up-regulating the BDNF-TrkB pathway function by local infusion of rhBDNF or 7,8-DHF. Subchronic intraperitoneal administration of 7,8-DHF also successfully reversed ELS-induced neurogenesis reduction in adult mice. Taken together, our results suggest that BDNF and its TrkB receptor are causally involved in the cognitive impairment caused by early-life stress, which can serve as the treatment targets for the cognitive deficits of stress-related psychiatric disorders.

The BDNF-TrkB pathway is closely associated with cognitive functions. For instance, hippocampus-dependent learning and memory could be impaired by downregulating endogenous BDNF via genetic knockdown [27, 28] or inhibition of TrkB receptor using ANA-12 [49, 55]. Consistent with these findings, we observed that BDNF knockdown and local infusion of ANA-12 in DG significantly impaired spatial object recognition, mimicking the spatial memory deficits induced by early-life stress. To examine the possibility that the failure to recognize displaced objects in the SOR test results from general memory impairments, we assessed animals’ habituation by comparing the total probe time of objects between the acquisition and test phases and found that the total probe time in the test phase was significantly shorter as compared with the acquisition phase (repeated measures ANOVA, main effects of phase*, p*s < 0.024), with a lack of group by phase interactions, in 6 out of 8 experiments (Fig. S7). These results indicate that impaired SOR performances induced by early-life stress or BDNF-TrkB inhibition could not be attributed to a lack of habituation and may specifically reflect memory deficits of object locations. In contrast, hippocampus-dependent cognitive performance can be improved by enhancing the BDNF-TrkB pathway function via intra-hippocampal rhBDNF infusion [48, 56] or repeated systemic administration of 7,8-DHF [33]. In line with these results, we observed significant cognition-improving effects in control mice receiving local infusion of rhBDNF and 7,8-DHF in DG and suchronic (not acute) intraperitoneal treatment of 7,8-DHF. Our results therefore provide confirmatory evidence that the BDNF-TrkB pathway, especially in DG, is indeed causally involved in spatial memory.

It is worth noting that we did not observe spatial working memory deficits in the Y-maze spontaneous alternation test following the LBN paradigm, which is inconsistent with our previous findings [10], in which control mice received intraperitoneal vehicle injections. It is also possible that the effect of the LBN procedure on this task are not convergent across studies. Future studies using alternative behavioral paradigms (e.g., the delayed match-to-sample or non-match-to-sample task) are warranted to clarify whether ELS leads to spatial working memory deficits.

Does the BDNF-TrkB pathway mediate ELS-induced cognitive deficits? Although it has been consistently shown that ELS decreases hippocampal BDNF expression levels [40, 41] and impairs cognition (e.g. [11, 43, 44]), to our knowledge, the role of the BDNF-TrkB pathway in ELS-induced cognitive deficits has not been empirically tested. Activating the TrkB receptor via 7,8-DHF-related treatment has been reported to rescue cognitive deficits in animal models of Alzheimer’s disease [31, 57, 58]. As for stress-induced cognitive deficits, one earlier study reported that 14-day hippocampal BDNF infusion before chronic immobilization stress could protect adult rats from stress-induced deficits in spatial learning and memory [59]. 7,8-DHF has also been found to prevent deficits in spatial learning in the Morris water maze induced by 2-h immobilization stress [60]. Here, for the first time, our acute experiments of local rhBDNF infusion and 7,8-DHF administration show that upregulating the BDNF-TrkB pathway restores ELS-induced spatial memory deficits, providing direct evidence that the BDNF-TrkB pathway is causally involved in ELS-induced cognitive deficits. More importantly, subchronic treatment of 7,8-DHF restored cognitive deficits three days after the last drug treatment. This long-lasting therapeutic effect rules out acute upregulation of the BDNF-TrkB functionality and might arise from successful reversal of ELS-induced molecular or plasticity alterations beyond the pathway itself, e.g., neurogenesis.

Besides cognitive functions, we investigated the involvement of the BDNF-TrkB pathway in ELS-induced reduction of DG neurogenesis. Divergent results have been reported in the effects of the LBN paradigm on DG neurogenesis, depending on animal age and neurogenesis markers. Neurogenesis has been found to be increased at PND10 [61] and decreased in adulthood in LBN-treated rodents [61–64]. Neurogenesis reduction in adult animals exposed to LBN is more pronounced in Brdu^+^ cells (indicative of neuronal survival) than DCX^+^ and Ki-67^+^ cells (indicative of proliferation and differentiation) [61–63]. Here we found that LBN significantly reduced adult neurogenesis in DG, measured by the number of DCX^+^ and Ki-67^+^ cells. The stronger stress effects on proliferation and differentiation in our study may arise from differences in mouse genetic backgrounds (C57BL/6N in our study vs. C57BL/6J in previous studies [61, 62]; C57BL/6N may be more sensitive to stress effects [65]) and/or LBN stress procedures (the stressed group was given 1/4 bedding materials in our study vs. 1/2 bedding materials in other studies [61–63] of the control group). As for the relationship between adult neurogenesis and short-term spatial memory (i.e., SOR test), previous studies have reported divergent results, with LBN-induced spatial memory deficits either related or unrelated to adult neurogenesis changes [61, 62]. This issue is beyond the scope of our study, as we aim to investigate the involvement of the BDNF-TrkB system in LBN-induced alterations in short-term spatial memory and adult neurogenesis. Future studies are warranted to empirically test their causal relationships. Finally, it is well-established that neurogenesis in DG is regulated by the BDNF-TrkB pathway [47]. Consistent with this notion, we observed that local BDNF knockdown in DG significantly reduced the number of DCX^+^ and Ki-67^+^ cells. It has also been shown that activating TrkB receptor via intraperitoneal injection of 7,8-DHF strongly promotes neurogenesis in control mice [47], mice exposed to moderate traumatic brain injury [66], and in depressive vulnerable rats [67]. Here we observed that, several days after the last treatment, chronic administration of 7,8-DHF still successfully reversed ELS-induced neurogenesis reduction. Collectively, our findings provide evidence that the BDNF-TrkB pathway underlies the effects of ELS on DG neurogenesis and serves as potential therapeutic targets to treat stress-induced neurogenesis reduction.

To conclude, our findings are the first to show the causal involvement of the BDNF-TrkB pathway on ELS-induced hippocampus-dependent spatial memory loss and hippocampal neurogenesis reduction. More importantly, our results of subchronic systemic administration of 7,8-DHF highlight the BDNF-TrkB pathway as the potential therapeutic targets for the treatment of cognitive deficits in stress-related psychiatric disorders. Currently, clinical drug development of 7,8-DHF faces several challenges, e.g., low oral bioavailability (see [32] for a review), but also makes great progress in preclinical studies (e.g. [57]). Future studies are warranted to better understand the mechanisms of 7,8-DHF in the prevention of early-life stress-induced cognitive deficits and examine the therapeutic effects of novel 7,8-DHF-related drugs.

## Supplementary information

Supplemental Information
