# Supplementary material for: The causal involvement of the BDNF-TrkB pathway in dentate gyrus in early-life stress-induced cognitive deficits in male mice
Source: Transl Psychiatry. 2023 May 24;13:173. doi: 10.1038/s41398-023-02476-5 (PMC10209152; doi:10.1038/s41398-023-02476-5)
Supplement: Supplementary file 1 — Supplemental Information [file 41398_2023_2476_MOESM1_ESM.docx]

**Supplemental Information for:**

**The causal involvement of the BDNF-TrkB pathway in dentate gyrus in early-life stress-induced cognitive deficits in male mice**

Ya-Xin Sun, Yun-Ai Su, Qi Wang, Jia-Ya Zheng, Chen-Chen Zhang, Ting Wang, Xiao Liu, Yu-Nu Ma, Xue-Xin Li, Xian-Qing Zhang, Xiao-Meng Xie, Xiao-Dong Wang, Ji-Tao Li, Tian-Mei Si

**Supplementary Figures**


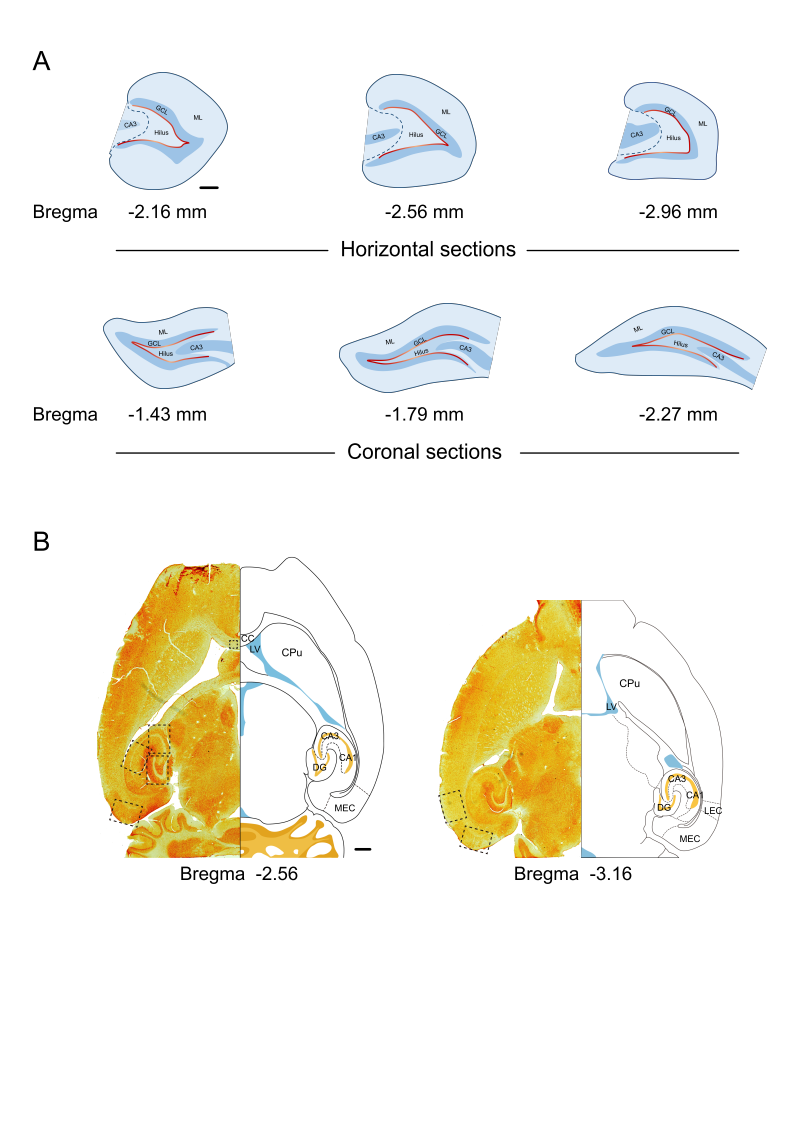


**Fig. S1 Schematic illustration of the laminated structure of dorsal DG and regions of interest analyzed in adult mice.** (A) Schematic of the dorsal DG showing the regions of interest in horizontal (upper row) and coronal (lower row) sections. The red line represents the length of granule cell layer. Scale bar = 200 µm. (B) Schematic illustration of subregions analyzed in mouse hippocampus and entorhinal cortex. Scale bar = 500 µm. CC, corpus callosum; CPu, caudate putamen; DG, dentate gyrus; LEC, lateral entorhinal cortex; LV, lateral ventricle; MEC, medial entorhinal cortex.


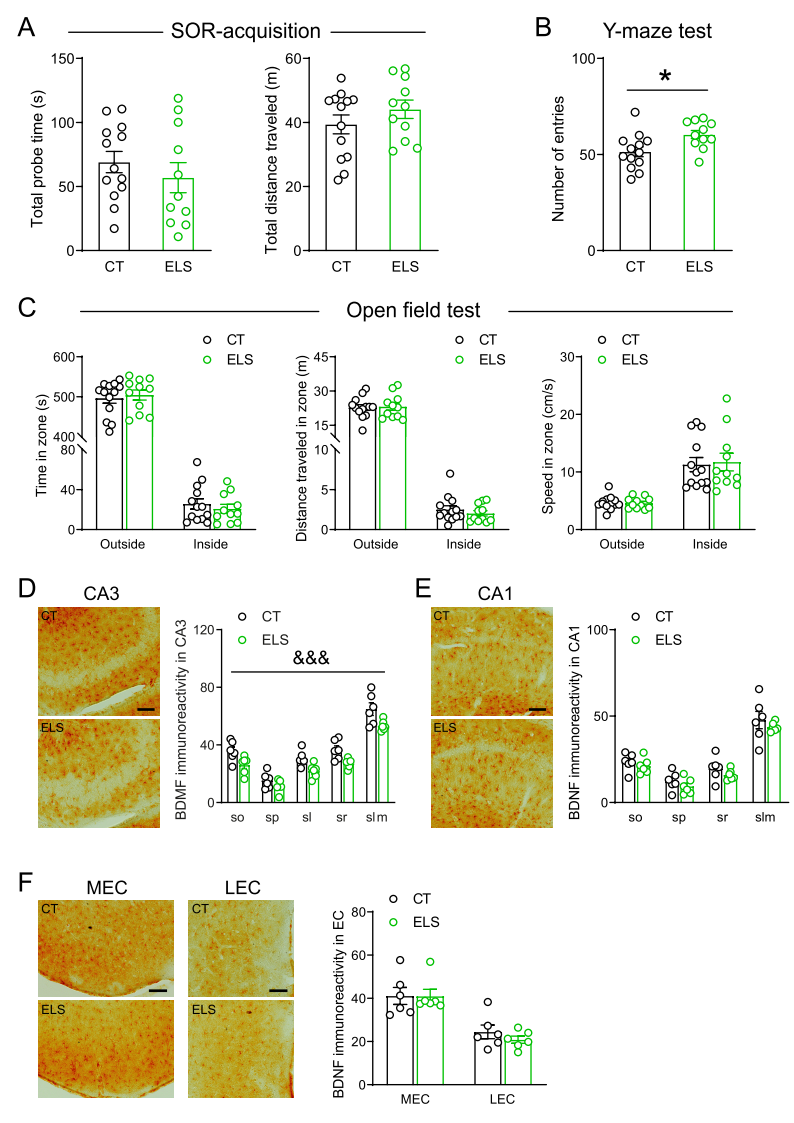


**Fig. S2 Additional results of the effects of early-life stress on cognitive and anxiety-like behaviors in adult mice.** (A) During the acquisition phase of the SOR test, control and stressed mice spent similar time interacting with two objects and traveled similar distance in the box. (B) In the Y-maze test, ELS significantly increased the number of entries to three arms. (C) In the open field test, ELS did not affect the time, distance traveled, or speed in the outside and inside zones. (D-F) Immunostaining revealed that ELS significantly reduced the protein levels of BDNF in the CA3 (D), not in the CA1 (E) or EC (F) in adult male mice. Scale bar = 100 µm. CT, control; EC, entorhinal cortex; LEC, lateral entorhinal cortex; MEC, medial entorhinal cortex; ELS, early-life stress; sl, stratum lucidum; slm, stratum lacunosum-moleculare; so, stratum oriens; SOR, spatial object recognition; sp, stratum pyramidale; sr, stratum radiatum. **p* < 0.05, unpaired *t* test; ^&&&^ *p* < 0.001, the main effect of stress.


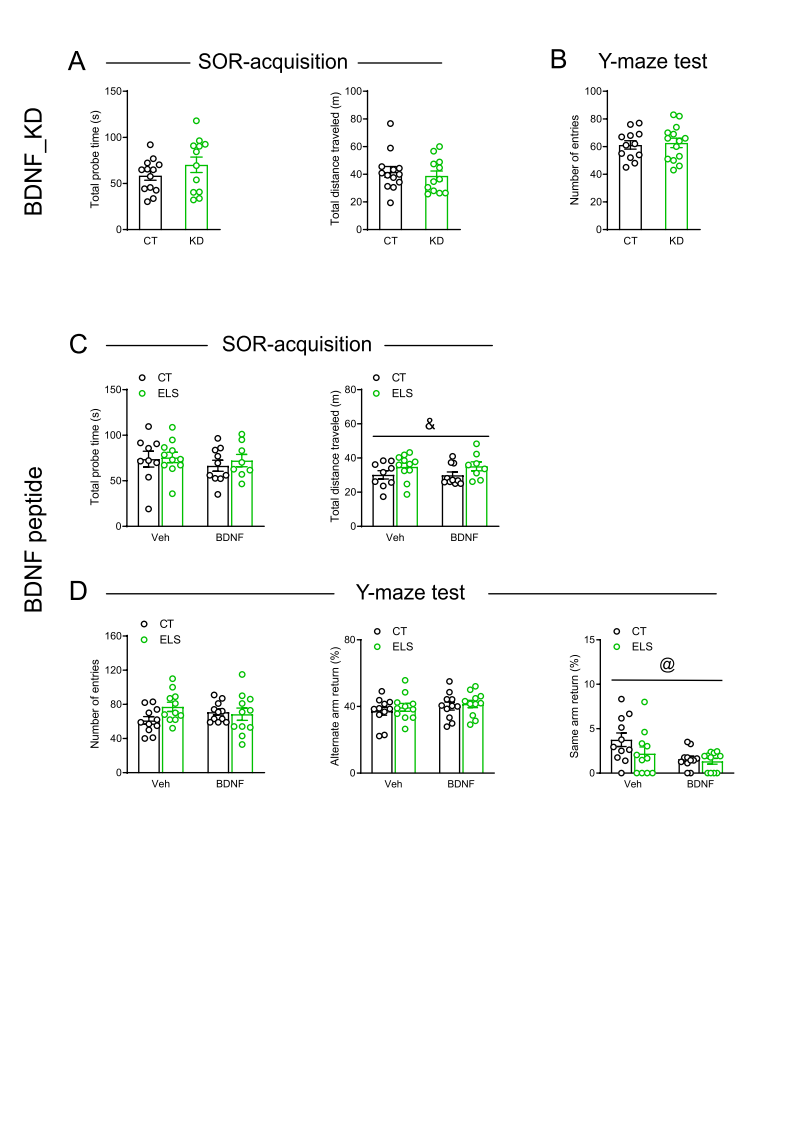


**Fig. S3 Additional results of the section “BDNF in the DG mediated early-life stress-induced spatial memory impairment”.** (A) During the acquisition phase of the SOR test, control and BDNF_KD mice showed comparable time interacting with two objects and traveled distance in the box. (B) In the Y-maze test, BDNF knockdown in the DG did not affect the number of entries to three arms. (C) During the acquisition phase of the SOR test, there were no main effects of rhBDNF in the total probe time or total distance traveled, whereas ELS significantly increased the total distance traveled, without affecting the total probe time. (D) In the Y-maze test, the total number of entries (left) and AAR ratio (middle) were not affected by ELS and rhBDNF. SAR ratio (right) was not significantly altered by ELS and was significantly reduced by rhBDNF. AAR, alternate arm return; BDNF, brain-derived neurotrophic factor; CT, control; ELS, early-life stress; KD, knockdown; SA, spontaneous alternation; SAR, same arm return; SOR, spatial object recognition; Veh, vehicle. ^&^ *p* < 0.05, the main effect of stress; ^@^ *p* < 0.05, the main effect of BDNF.


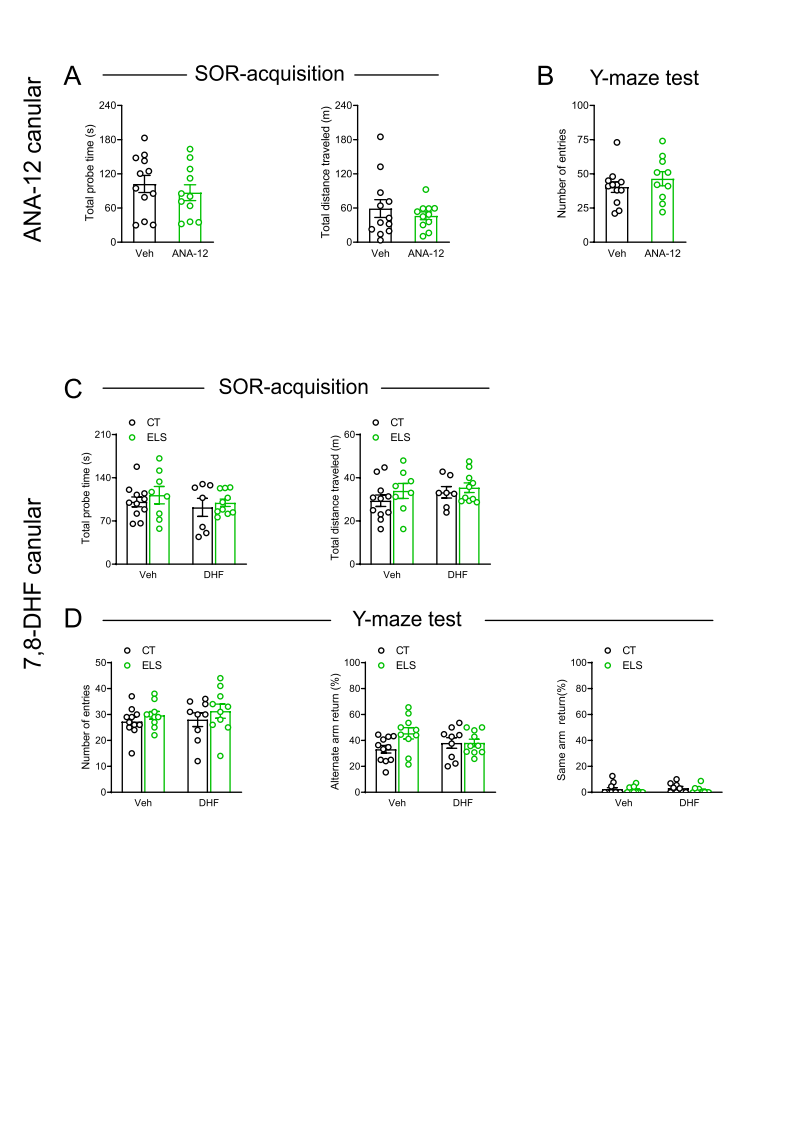


**Fig. S4 Additional results of the section “TrkB Receptor in DG Mediated Early-Life Stress-Induced Spatial Memory Loss”.** (A) During the acquisition phase of the SOR, control and ANA-12-treated mice showed comparable time interacting with two objects and traveled distance in the box. (B) In the Y-maze test, local ANA-12 injection in the DG did not affect the number of entries to three arms. (C) During the acquisition phase of the SOR, ELS and local 7,8-DHF injection did not significantly influence the total probe time or total distance traveled. (D) In the Y-maze test, the total number of entries (left), AAR ratio (middle), and SAR ratio (right) were not affected by ELS or 7,8-DHF. AAR, alternate arm return; CT, control; DHF, 7,8-dihydroxyflavone; ELS, early-life stress; SA, spontaneous alternation; SAR, same arm return; SOR, spatial object recognition; Veh, vehicle.


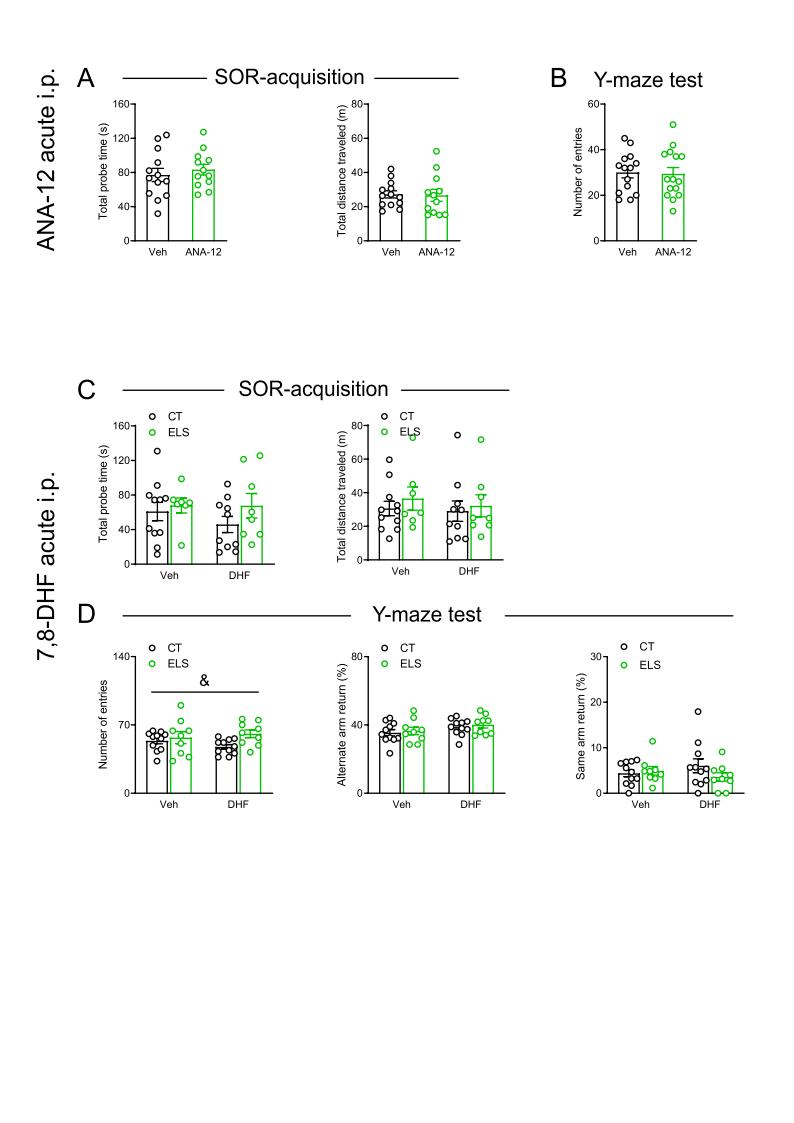


**Fig. S5 Additional results of the section “Acute Systemic Administration of 7,8-DHF Reversed Stress-Induced Spatial Memory Loss”.** (A) During the acquisition phase of the SOR test, control and ANA-12-treated mice showed comparable time interacting with two objects and traveled distance in the box. (B) In the Y-maze test, acute intraperitoneal ANA-12 injection did not affect the number of entries to three arms. (C) During the acquisition phase of the SOR test, ELS and acute 7,8-DHF injection (i.p.) did not significantly influence the total probe time or total distance traveled. (D) In the Y-maze test, compared with control mice, stressed mice showed increased number of entries (left) into three arms, whereas AAR ratio (middle) and SAR ratio (right) were not affected. AAR, alternate arm return; CT, control; DHF, 7,8-dihydroxyflavone; ELS, early-life stress; i.p., intraperitoneal; SA, spontaneous alternation; SAR, same arm return; SOR, spatial object recognition; Veh, vehicle. ^&^ *p* < 0.05, the main effect of stress.


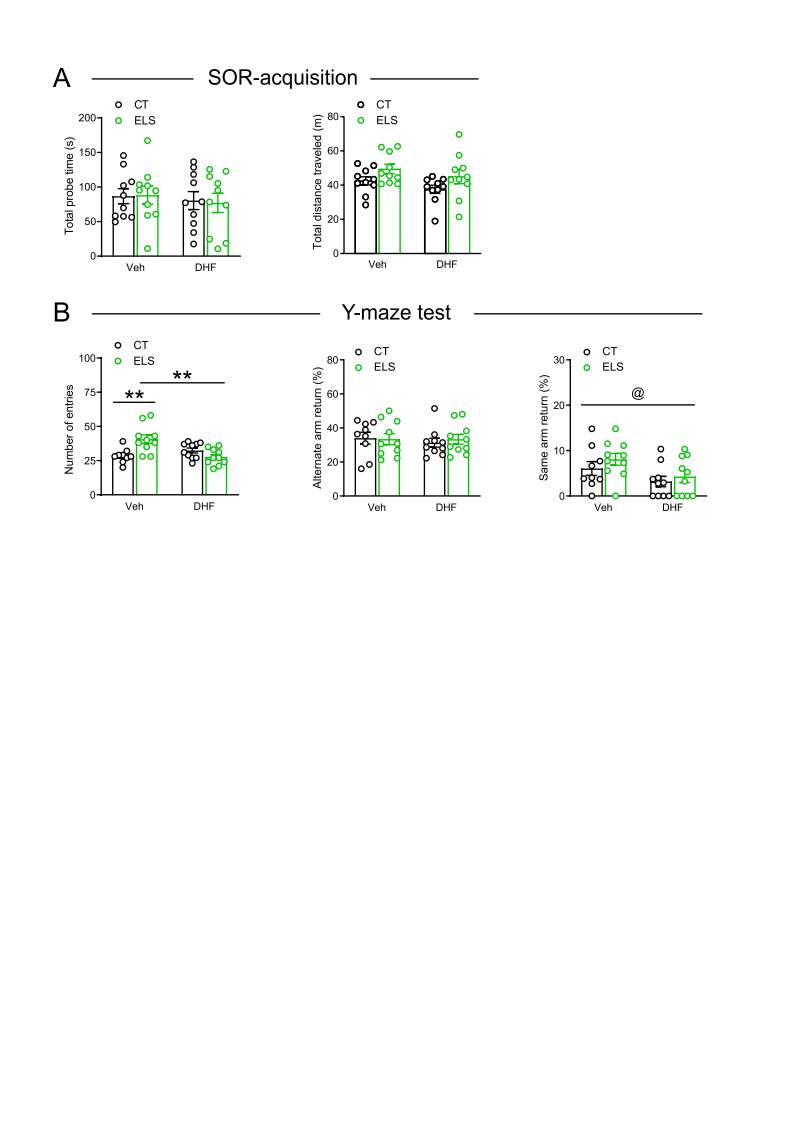


**Fig. S6 Additional results of the section “Subchronic Systemic Administration of 7,8-DHF Reversed ELS-induced Spatial Memory Deficits and Adult neurogenesis reduction”.** (A) During the acquisition phase of the SOR test, ELS and subchronic 7,8-DHF injection (i.p.) did not significantly influence the total probe time or total distance traveled. (B) In the Y-maze test, the increased number of entries (left) induced by ELS was normalized by subchronic 7,8-DHF injection (i.p.). AAR ratio (middle) was not affected by ELS or 7,8-DHF, while SAR ratio (right) was significantly decreased by 7,8-DHF. AAR, alternate arm return; CT, control; DHF, 7,8-dihydroxyflavone; ELS, early-life stress; i.p., intraperitoneal; SA, spontaneous alternation; SAR, same arm return; SOR, spatial object recognition; Veh, vehicle. ***p* < 0.01, Tukey’s *post hoc* test; ^@^ *p* < 0.05, the main effect of drug.


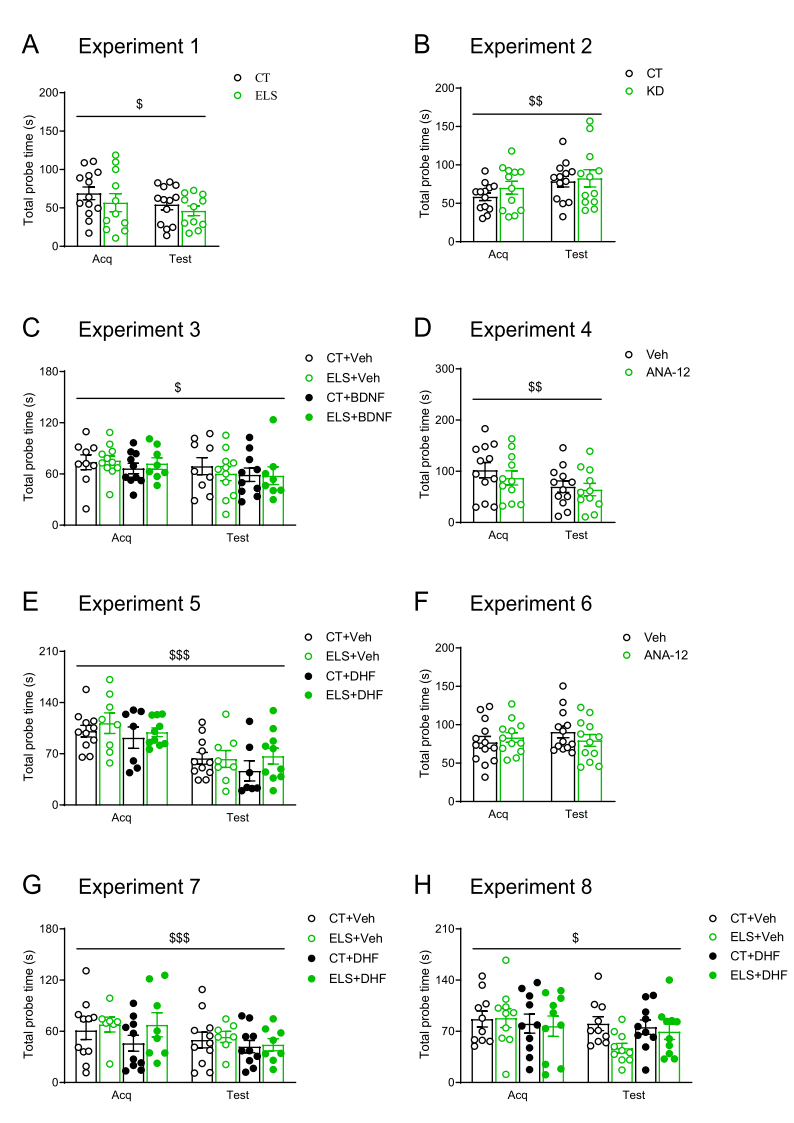


**Fig. S7 The total probe time of objects in the acquisition and test phases in spatial object recognition in Experiment 1-8 (A-H).** Acq, acquisition; BDNF, brain-derived neurotrophic factor; CT, control; DHF, 7,8-dihydroxyflavone; ELS, early-life stress; KD, knockdown; Veh, vehicle. ^$^ *p* < 0.05, ^$$^ *p* < 0.01, ^$$$^ *p* < 0.001, the main effect of phase, repeated measures ANOVA.
